# High-fat diet impairs ferroptosis and promotes cancer invasiveness via downregulating tumor suppressor ACSL4 in lung adenocarcinoma

**DOI:** 10.1186/s13062-021-00294-7

**Published:** 2021-05-31

**Authors:** Yixiang Zhang, Songyu Li, Fengzhou Li, Changsheng Lv, Qing-kai Yang

**Affiliations:** 1 Department of Thoracic Surgery, The First Affiliated Hospital of Dalian Medicine University, No. 222 Zhongshan Road, Liaoning 116000 Dalian, China; 2 Department of Oncology, Institute of Cancer Stem Cell, Dalian Medical University, 9 Western Lvshun South Road, Liaoning 116044 Dalian, China

**Keywords:** Long-chain acyl-CoA synthetase-4, Ferroptosis, Lung adenocarcinoma, High-fat diet

## Abstract

**Background:**

Long-chain acyl-CoA synthetase-4 (ACSL4) is involved in fatty acid metabolism, and aberrant ACSL4 expression could be either tumorigenic or tumor-suppressive in different tumor types. However, the function and clinical significance of ACSL4 in lung adenocarcinoma remain elusive.

**Results:**

ACSL4 was frequently downregulated in lung adenocarcinoma when analyzing both the TCGA database and the validation samples, and the lower ACSL4 expression was correlated with a worse prognosis. Using gene set enrichment analysis, we found that high ACSL4 expression was frequently associated with the oxidative stress pathway, especially ferroptosis-related proteins. *In vitro* functional studies showed that knockdown of ACSL4 increased tumor survival/invasiveness and inhibited ferroptosis, while ACSL4 overexpression exhibited the opposite effects. Moreover, high-fat treatment could also inhibit erastin-induced ferroptosis by affecting ACSL4 expression. The anti-tumor effects of ferroptosis inducers and the anti-ferroptosis effects of the high-fat diet were further validated using the mouse xenograft model.

**Conclusions:**

ACSL4 plays a tumor-suppressive role in lung adenocarcinoma by suppressing tumor survival/invasiveness and promoting ferroptosis. Our study provided a theoretical reference for the application of ferroptotic inducers and dietary guidance for lung adenocarcinoma patients.

**Supplementary Information:**

The online version contains supplementary material available at 10.1186/s13062-021-00294-7.

## Background

Long-chain acyl-CoA synthetase-4 (ACSL4), a member of the long-chain acyl-coenzyme synthetase (ACSL) family, is involved in the biosynthesis and catabolism of fatty acids. There are five different isoenzymes in the ACSL family, ACSL1, ACSL3, ACSL4, ACSL5, and ACSL6. ACSLs prefer fatty acids with chain lengths of 12 to 20 carbons as substrates [1], and ACSLs are also the response gene for peroxisome proliferator-activated receptor gamma (PPARγ), which mediates the lipid metabolism and regulates caloric absorption [2]. The dysregulation of ACSL4 is associated with a variety of lipid metabolism diseases. Different from other family members, ACSL4 catalyzes the synthesis of arachidonic acid (AA) into arachidonic acid coenzyme A, which participates in the synthesis of membrane phospholipids. ACSL4 enriched long polyunsaturated ω6 fatty acids in cell membranes and dictated ferroptosis sensitivity by shaping cellular lipid composition [3, 4]. Ferroptosis, which is different from apoptosis and necrosis, is closely related to the disturbance of iron-dependent lipid peroxides, and these accumulated lipid reactive oxygen species (L-ROS) could lead to ferroptotic cell death. Previous studies found that inducers of ferroptosis could inhibit tumor growth and kill cancer cells [5–7], and activation of ferroptosis becomes a novel and effective way of cancer intervention. On the other hand, some studies showed that ACSL4 is associated with the development and progression of multiple cancers, including breast cancer [8, 9], colorectal cancer [10], and hepatocellular carcinoma [11]. As ACSL4 could exhibit either tumor-suppressive or tumor-promoting functions, it is worth specifying the role of ACSL4 based on the specific cancer type, including lung adenocarcinoma where ACSL4 is rarely studied.

Lung adenocarcinoma is the main type of lung cancer and accounts for around 30 % of all newly diagnosed lung cancer worldwide [12]. Due to the early metastasis and recurrence of lung adenocarcinoma, the 5-year survival rate is below 30 % [13]. Identifying genes that are aberrantly expressed helps understanding the initiation and progression of lung adenocarcinoma. In this study, we used the gene expression data from the TCGA database to characterize the patient prognosis stratified based on ACSL4 expression and understand the ACSL4-related pathways in lung adenocarcinoma. Furthermore, we investigated the cellular function of ACSL4 and its impact on ferroptosis using *in vitro* cell line models. Lastly, we studied the effect of the high-fat diet on erastin-induced ferroptosis using both cell line and mouse xenograft models.

## Methods

### Database analysis for ACSL4 expression and patient prognosis

We used GEPIA to analyze ACSL4 expression and evaluate its relation to cancer patient survival. GEPIA was an interactive web server for estimating the mRNA expression data from 9,736 tumors and 8,587 normal samples in the Cancer Genome Atlas (TCGA) and Genotype-Tissue Expression (GTEx) dataset projects (http://gepia.cancer-pku.cn/) [14–18]. The expression of ACSL4 in 33 types of cancers and the matched normal samples was identified from the GEPIA database. The mRNA expression in cancer tissue compared to normal tissue was obtained, and P < 0.01 and |Log_2_FC|>1 were set as the screening criteria for differentially expressed genes. The expression profiles of ACSL4 isoforms in different types of cancers were identified and presented as boxplots.

The Kaplan-Meier Plotter (http://kmplot.com) is an online database that comprises gene expression information and clinical outcome parameters of breast cancer, lung cancer, liver cancer, ovarian cancer, and gastric cancer [19]. The prognostic value of ACSL4 in lung adenocarcinoma was determined by Kaplan-Meier analysis using the Kaplan-Meier-plotter online software.

### Gene set enrichment and pathway analysis

To better understand the underlying molecular role of ACSL4 in lung adenocarcinoma, we further analyzed data using TCGA database. The clinical and gene expression data of 524 patients with lung adenocarcinoma were obtained from TCGA (https://tcga-data.nci.nih.gov/tcga/). We used R software to perform molecular role analysis of TCGA database. Pathway enrichment analyses were performed using gene set enrichment analysis (GSEA). Genes related to oxidative stress were selected using the Gene Ontology (GO) database with the search term ‘response to oxidative stress’ (http://www.geneontology.org/). We explored the biological functions of the gene list and screened for genes involved in cellular oxidative stress. Patients were divided into high ACSL4 expression group (n = 262) and low ACSL4 expression group (n = 262), and the median ACSL4 expression level was used to separate the high and low expression of ACSL4 patients. Relationships between ACSL4 expression levels and genes involved in cellular oxidative stress were displayed as a heat map. The KEGG pathway database was a collection of manually drawn pathway maps of molecular interactions. To clarify the regulatory relationship between ACSL4 expression and ferroptosis-related gene expression. Pathway network analysis was based on the signal pathway of ferroptosis in the KEGG database, and the interaction network diagram of pathway research was constructed (https://www.genome.jp/keggbin/show_pathway?map04216).

### Antibodies and other reagents

Primary antibodies against ACSL4 and β-actin were purchased from Proteintech (Hubei Province, China). Erastin, a compound that could induce ferroptosis in mammalian cells, was purchased from Invitrogen (California, USA). The SP-900 general immunohistochemical kit was purchased from ZSGB-BIO (Beijing, China). Cell/tissue protein extraction kits were purchased from Tiangen Biotech (Beijing, China). Other chemicals and reagents such as MTT, sodium dodecyl sulfate, Acryl&Bis premixed powder, BCA protein quantification kit, and horseradish peroxidase (HRP) substrate luminescent solution were purchased from Sigma (St. Louis, MO, USA), Vetec (St. Louis, MO, USA) and Biological Industries (Kibbutz Beit Haemek, Israel).

### Cell culture and tissue samples

In order to investigate the functional changes of ACSL4, experiments were performed with lung adenocarcinoma cancer cell lines. The cell lines A549, H322, H1299, and H460 were purchased from American Type Culture Collection (ATCC, Manassas, VA, USA). All four cell lines were maintained in DMEM containing 10 % FBS and 1 % penicillin–streptomycin at 37 °C under a humidified atmosphere of 5 % CO_2_. Human lung adenocarcinoma tissue samples and corresponding adjacent tissue samples were obtained from 10 patients undergoing thoracoscopic treatment from the first affiliated hospital of Dalian Medical University. This study was approved by the Ethics Committee of the First Affiliated Hospital of Dalian Medical University. All patients who underwent surgical resection have signed informed consent to our Hospital. ACSL4 expression was assessed by immunohistochemical staining using an anti-ACSL4 antibody and quantitative real-time PCR.

### Protein extraction and western blot analysis

The cells were collected in a microcentrifuge tube at optimum cell culture time. Protein extraction was performed according to our previous publication [20]. The concentration of total protein was quantified by the BCA protein assay kit (Sigma, St. Louis, MO, USA). The proteins were separated with 10 % sodium dodecyl sulfate-polyacrylamide gel electrophoresis (SDS-PAGE) and transferred onto polyvinylidene fluoride membranes. The membrane was incubated with specific primary and secondary antibodies. Afterward, the protein bands were detected using an enhanced chemiluminescence system.

### Plasmid construction, RNA interference, and transfection

A plasmid containing the full-length ACSL4 ORF and small interfering RNAs (siRNAs) was created and synthesized by Gene Pharma (Shanghai, China). Targeting sequences for shRNAs are as follows: h1 5’-AGCAGAGATATCTTGCTTT-3’, sh2 5’-TAGATATCAGTTGTGTTAA-3’, sh3 5’-TGCAATCTGTTACTGTTTA-3’, and sh4 5’-CCGATGGATGTTTACAGAT-3’. The effect of gene silencing was evaluated using western blot analysis. Sh2 was used because of the most obvious silent effect. All procedures were performed according to the manufacturer’s protocol.

### Cell viability assay

The viability of the cells was determined by MTT assay, which is based on the conversion of MTT to formazan crystals by mitochondrial dehydrogenases. Cells were seeded at a density of 1 × 10^4^ cells per well in 96 well plates, and they were then transfected with shACSL4, oeACSL4, or control PBS at the indicated doses. The cells were incubated with MTT for 3 h at 5 % CO_2_ and 37 °C. The absorbance was determined at 490 nm by an enzyme-linked immunosorbent assay reader.

### Transwell assays

Transwell assays were performed to assess cell migration and invasion ability using a transwell chamber with pore size of 8.0 μm. Cells were serum-starved overnight at 37 °C in DMEM prior to treatment. 1 × 10^5^ cells/well were placed in the upper chamber containing DMEM with 10 % FBS. After 24 h of incubation, the cells were rinsed with PBS. Cells were fixed with 4 % paraformaldehyde solution for 10 min and room temperature, stained with 0.1 % crystal violet solution for 10 min at room temperature, and counted by a microscope using 5 randomly selected fields of view at a 200x magnification.

### Wound healing assay

Cell migration was assayed using the wound healing assay. Cells were transfected with siACSL4, oeACSL4, or control PBS or treated with Erastin or Ferrostatin-1, and they were then plated in 6-well plates to create a confluent monolayer. A linear scratch or wound was made across the confluent monolayer using a fine 100 µL pipette tip. The spacing of the gap was photographed by an inverted microscope at 0 h, 24 h, and 48 h.

### Lipid peroxidation assay

We mainly used liperfluo to monitor the ferroptosis [21–23]. Liperfluo was used to test the lipid peroxidase (LPO) level in cells treated with erastin following the manufacturer’s protocol. Liperfluo emits intense fluorescence by lipid peroxide-specific oxidation in organic solvents such as ethanol. Among fluorescent probes that detect reactive oxygen species (ROS), liperfluo is the only compound that can specifically detect lipid peroxides. It can easily be applied to lipid peroxide imaging by a fluorescence microscopy and a flow cytometric analysis for living cells. We add liperfluo (final concentration of 20 µM) according to the manufacturer**´**s protocol, followed by collecting cells with PBA and analyzing by flow cytometer.

### Fluorescence-activated cell sorting (FACS) analysis of cell apoptosis

Apoptosis was detected with a fluoresceinisothiocyanate (FITC) Annexin V Apoptosis Detection Kit (Sigma), according to the manufacturer’s instructions. Approximately 2 × 10^6^ cells that were treated with the indicated condition were washed twice with PBS. The cells were then incubated in 0.2 mL of binding buffer containing 5 µL of FITC-annexin V in the dark for 10 min at room temperature. Subsequently, 10 µL of propidium iodide was added, and samples were analyzed with a flow cytometer (BD Accuri C6 plus).

### High-fat treatment

For the high-fat treatments, cells were cultured with DMEM (400 µmol/L of the saturated free fatty acid palmitate [16:0]) for 48 h at 37 °C with 5 % CO_2_, according to the manufacturer’s protocol. Acid palmitate was obtained from Xian Kunchuang Science and Technology Develop Co. Ltd (Xian, China) [24, 25]. Mice were assigned into different groups and fed with either standard feed (StF) or high-fat diet (HFD). High-fat treatment groups were fed a high-fat feed (as follows: standard feed; lard (fat), 30–40 %; pig bile salt, 3.5 %) for two weeks.

### Xenograft mouse model and tumor processing

Twelve nude mice (5–6 weeks old, 16–18 g weight) were managed at SPF Laboratory Animal Center at Dalian medical university in strict accordance with the guidelines by the U.S. National Institutes of Health Guide for the Care and Use of Laboratory Animals. To assess the effect of high-fat treatment on lung adenocarcinoma growth *in vivo*, the nude mice were randomly divided into four groups (ordinary diet + PBS group, n = 3; ordinary diet + erastin group, n = 3; high-fat diet + PBS group, n = 3; high-fat diet + erastin group, n = 3) and injected 2 × 10^6^ A549 cells on the abdominal region at both flanks. One week after the injection, four groups of mice were treated with erastin or PBS at 200 or 100 µg/mouse one time every 24 h by intra peritoneal injection for two consecutive weeks. Then all mice were terminated with ether anesthesia inhalation and humanely sacrificed. The tumor volume was measured with V = (width^2^× length)/2) and body weight was recorded. Tumor specimens were fixed in formalin and embedded in paraffin or directly stored at − 80 °C.

### Statistical analysis

P-values were calculated using a student’s t-test, spearman correlation analysis, or chi-square test as noted. P-values < 0.05 were considered statistically significant. For wound healing assay testing different treatments, the multiple comparisons of the student’s t-test were corrected using the Bonferroni method.

## Results

### ACSL4 was frequently downregulated and patients with low expression had a poor prognosis in lung adenocarcinoma

**Fig. 1:**
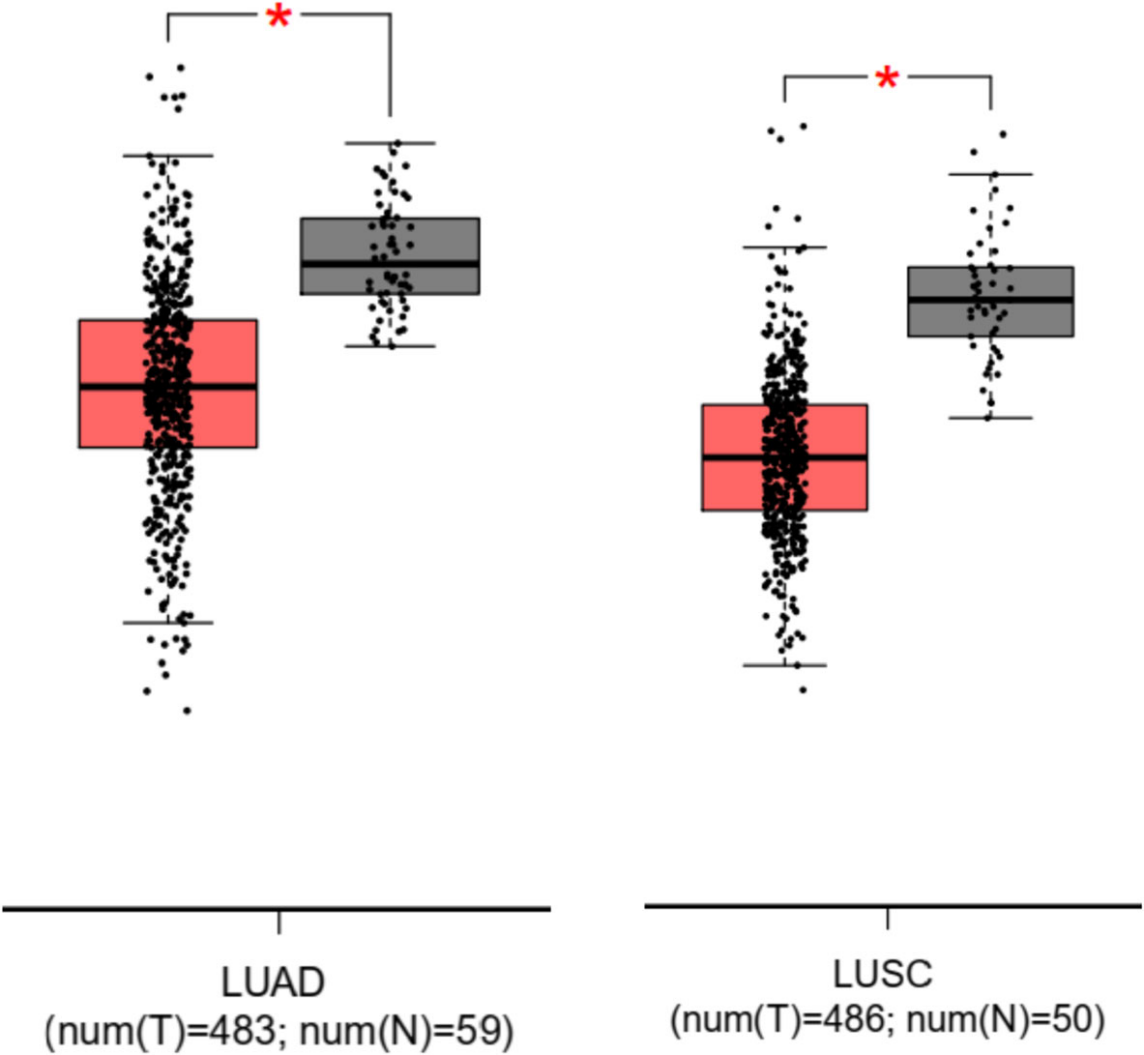
Aberrant ACSL4 expression in lung cancers. The ACSL4 expression data in the tumors (shown in red) and the corresponding normal tissues (shown in grey) were obtained from the TCGA database, and the number of tumor and normal samples (num (T) and num (N), respectively) was shown below each boxplot. The red asterisk represents p value < 0.05. LUAD, lung adenocarcinoma; LUSC, lung squamous cell carcinoma

**Fig. 2:**
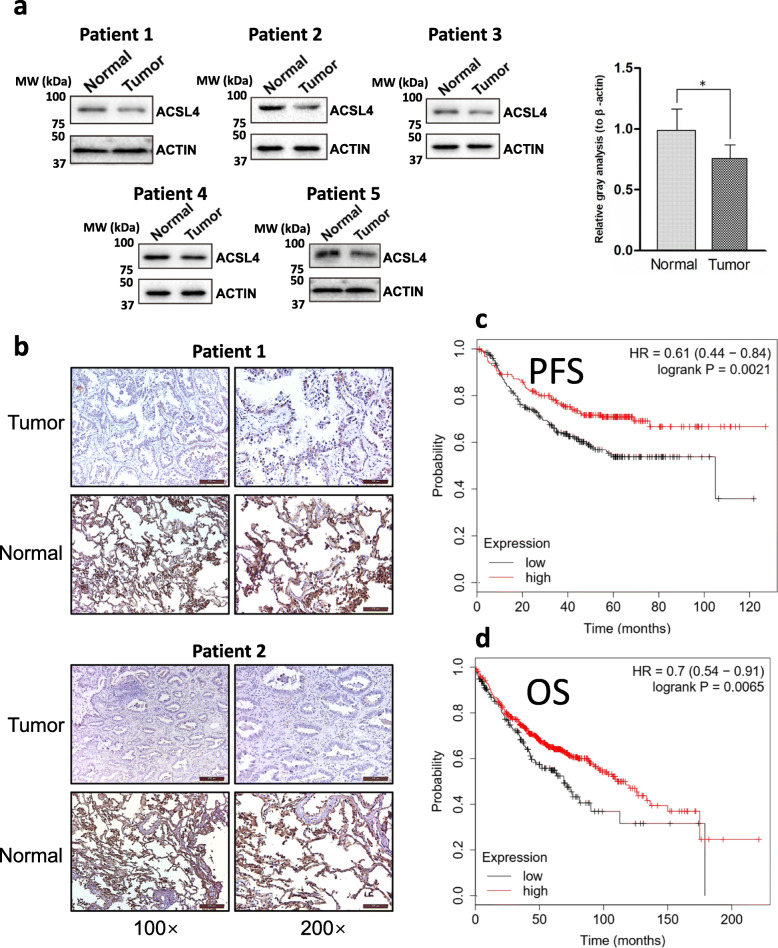
Low ACSL4 expression was associated with poor prognosis in lung adenocarcinoma. **a** The expression of ACSL4 in lung adenocarcinoma tissues and matched normal tissues was detected using Western blot. The samples were obtained from our hospital. Representative Western blot results were shown and ACTIN was used as the loading control. The quantification of the results (the intensity of ACSL4 divided by the intensity of ACTIN) was shown on the right. **b** The expression of ACSL4 in lung adenocarcinoma tissues was detected using immunohistochemistry. The samples were obtained from our hospital. **c-d** Kaplan-Meier analysis of progression-free survival (**c**) and overall survival (**d**) in lung adenocarcinoma patients. The patient survival data were obtained from the TCGA database (n = 524). The optimal ACSL4 expression level that was auto-selected by Kaplan Meier Plotter (see Method section for more details) was used as the cut-off to separate the high and low expression of ACSL4 patients. PFS, progression-free survival; OS, overall survival

We firstly assessed ACSL4 expression among 33 different types of cancers using the TCGA database, and several cancers demonstrated statistically significant differences in the ACSL4 expression level when compared with the corresponding normal control samples. Specifically, breast invasive carcinoma (BRCA), kidney chromophobe (KICH), kidney renal clear cell carcinoma (KIRC), kidney renal papillary cell carcinoma (KIRP), lung squamous cell carcinoma (LUSC), prostate adenocarcinoma (PRAD), and lung adenocarcinoma (LUAD) showed downregulation of ACSL4, while cholangial carcinom (CHOL), colon adenocarcinoma (COAD), and liver hepatocellular carcinoma (LIHC) had upregulated ACSL4 protein level (Fig. 1 and Fig. S1), which coincides with the previous findings that the specific role of ACSL4 in the tumor might vary according to the cancer type. As ACSL4 was rarely studied in lung adenocarcinoma, we then tried to validate the ACSL4 expression level in lung adenocarcinoma patient samples obtained from our hospital. ACSL4 showed significantly lower expression in most tumor samples using Western blot (Fig. 2a), and the result was further confirmed using immunohistochemistry (Fig. 2b). Intriguingly, the Kaplan-Meier survival analysis showed that lung adenocarcinoma patients with low ACSL4 expression had worse progression-free survival (PFS) and overall survival (OS) than those with high ACSL4 expression (Fig. 2c,d), suggesting the potential prognostic values of ACSL4 expression in lung adenocarcinoma.

### ACSL4 positively regulated ferroptosis in lung adenocarcinoma

**Fig. 3:**
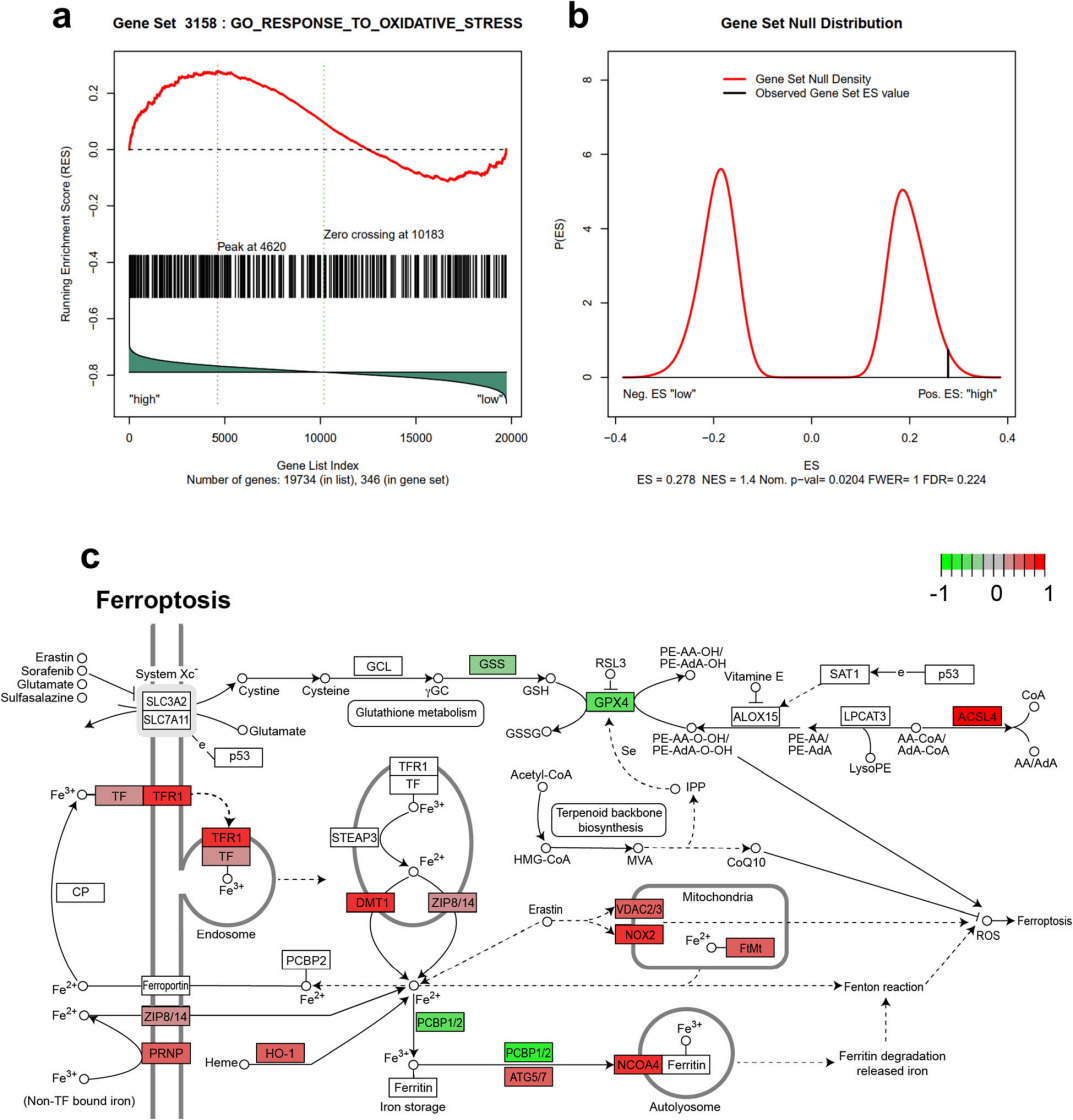
ACSL4 positively regulated ferroptosis in lung adenocarcinoma. **a** Gene set enrichment analysis (GSEA) revealed that oxidative stress-related genes were enriched in the high ACSL4 group. **b** The null distribution of the genes in the oxidative stress gene set. The false discovery rate was 22.4 % and the p value was 0.02. **c** Relationship between ACSL4 and genes in ferroptotic cell death signaling pathways by KEGG pathway enrichment analysis. The median ACSL4 expression level was used to separate the high and low expression of ACSL4 patients

Given the association between the ACSL4 expression level and patient prognosis, we next investigated the function of ACSL4 in lung adenocarcinoma. The gene expression data were obtained from 524 lung adenocarcinoma patients from the TCGA database and were separated into high ACSL4 expression group (n = 262) and low ACSL4 expression group (n = 262), using the median ACSL4 expression level as the cut-off point (See Method section for more details). Of note, GSEA and GO enrichment analyses revealed that oxidative stress-related genes were highly enriched in patients with increased ACSL4 expression (p = 0.02) (Fig. 3a,b), implying that ACSL4 might be involved in the regulation of the oxidative stress pathway. The oxidative stress-related genes that were enriched in the high ACSL4 group were listed in Table S1. Moreover, we found that ACSL4 expression was positively correlated with the upregulation of transferrin receptor protein 1 (TFR1), divalent metal transporter 1 (DMT1), prion protein (PRNP), Heme oxygenase-1 (HO-1), and NADPH oxidase 2 (NOX2), which have been reported to regulate cellular iron uptake, iron metabolism, or the generation of reactive oxygen species (ROS) (Fig. 3c). On the other hand, high ACSL4 expression was associated with downregulation of multiple proteins that promote intracellular iron storing/sequestering in ferritin or antioxidant activities, such as poly-(rC)-binding protein (PCBP), microtubule-associated proteins 1 A/1B light chain 3B (LC3), glutathione peroxidase 4 (GPX4), and glutathione synthetase (GSS) (Fig. 3c). Considering that all of these affected proteins were related to ferroptosis (a.k.a. iron-dependent cell death), high ACSL4 expression might be associated with elevated ferroptosis activity in lung adenocarcinoma, such as enhanced iron uptake, increased ROS generation, decreased iron chelation, and impaired antioxidant pathway.

### ACSL4 inhibited tumor cell survival, invasion, and migration and promoted ferroptosis in lung adenocarcinoma

**Fig. 4:**
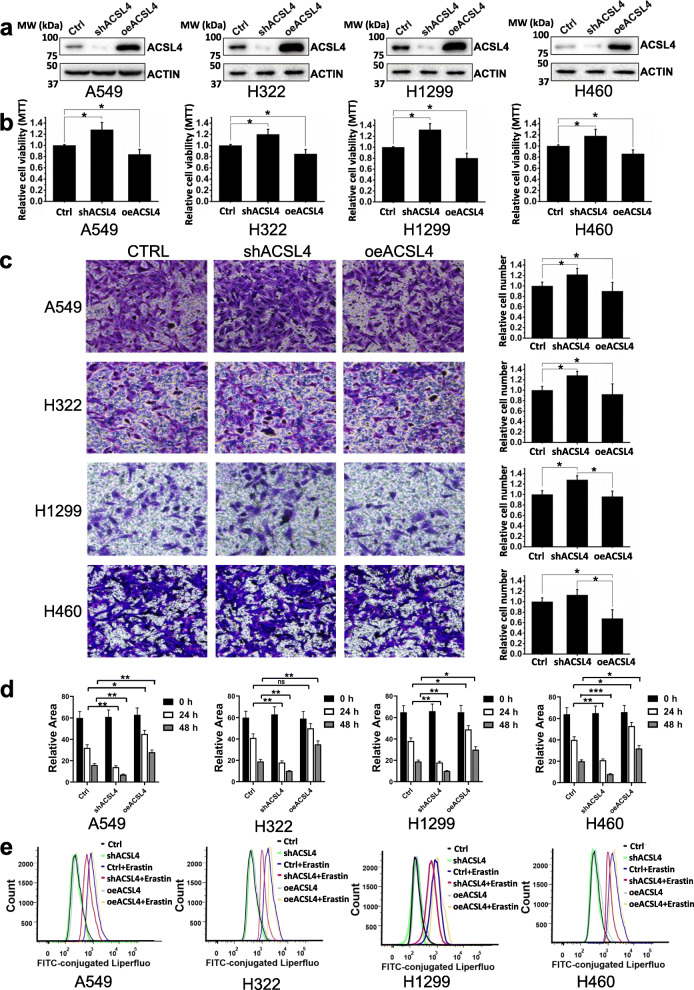
ACSL4 inhibited cell survival and tumor invasion/migration and promoted ferroptosis in lung adenocarcinoma. **a** The expression of ACSL4 in 4 different lung adenocarcinoma cell lines after ACSL4 knockdown (shACSL4) or overexpression (oeACSL4). Representative Western blot results were shown and ACTIN was used as the loading control. **b** Cell viability was analyzed by MTT in lung adenocarcinoma cells after ACSL4 knockdown or overexpression (n = 3). **c** Cell migration and invasion ability was analyzed by the transwell assay in lung adenocarcinoma cells after ACSL4 knockdown or overexpression. The quantification of the transwell assay for each cell line was shown on the right (n = 3). **d** Cell migration was analyzed by wound healing assay in lung adenocarcinoma cells after ACSL4 knockdown or overexpression (n = 3; *p < 0.05, **p < 0.01, ***p < 0.001). The multiple comparisons of student’s t-test (two-tailed) were corrected using the Bonferroni method. ns, not significant. **e** Liperfluo-based lipid peroxidation assay was used to monitor the ferroptosis levels in lung adenocarcinoma cells after ACSL4 knockdown, ACSL4 overexpression, and/or erastin treatment

As clinical and gene expression data indicated that high ACSL4 expression correlates with improved prognosis and enhanced ferroptosis in lung adenocarcinoma patients, we next investigated whether altering ACSL4 expression level could affect tumor growth and invasiveness in lung adenocarcinoma. We first knocked down (shACSL4) or overexpressed ACSL4 (oeACSL4) in 4 lung adenocarcinoma cell lines, including A549, H322, H1299, and H460 (Fig. 4a). ACSL4 knockdown cells had a higher number of viable cells while the oeACSL4 cells exhibited a significant growth inhibition when compared with the control cells (Fig. 4b). Moreover, both the transwell assay and wound healing assay indicate that knockdown of ACSL4 could enhance cell invasion and migration for all 4 lung adenocarcinoma cell lines (Fig. 4c,d, and Fig. S2), suggesting that ACSL4 acts as a tumor suppressor in lung adenocarcinoma. Due to its implications in ferroptosis, we further studied the effect of changed ACSL4 expression on ferroptosis. We coupled Liperfluo staining with flow cytometry to monitor the level of cellular lipid peroxides, which reflects the extent of ferroptosis. As shown in Fig. 4e, although the basal lipid peroxide levels were low and indistinguishable among cells with different ACSL4 expression levels, ACSL4 knockdown could reduce the erastin-induced ferroptosis while ACSL4 overexpression further exacerbated ferroptosis. Similar to ACSL4 overexpression, erastin treatment impaired cell migration, invasion, and survival in lung adenocarcinoma cells (Fig. S3); in contrast, ferroptosis inhibitor Ferrostatin 1 demonstrated the opposite effects, like those in ACSL4 knockdown cells (Fig. S3). We further confirmed that the majority of these treated cells were not died from apoptosis, (Fig. S4), implying that ferroptosis might be the modality of cell death. Overall, these results suggest that ACSL4 exhibits tumor suppressor roles in lung adenocarcinoma, and it could inhibit the growth and migration of the tumor cells and promote ferroptosis.

### High-fat treatment promoted cell survival, invasion, and migration and inhibited ferroptosis in lung adenocarcinoma by downregulating ACSL4

**Fig. 5:**
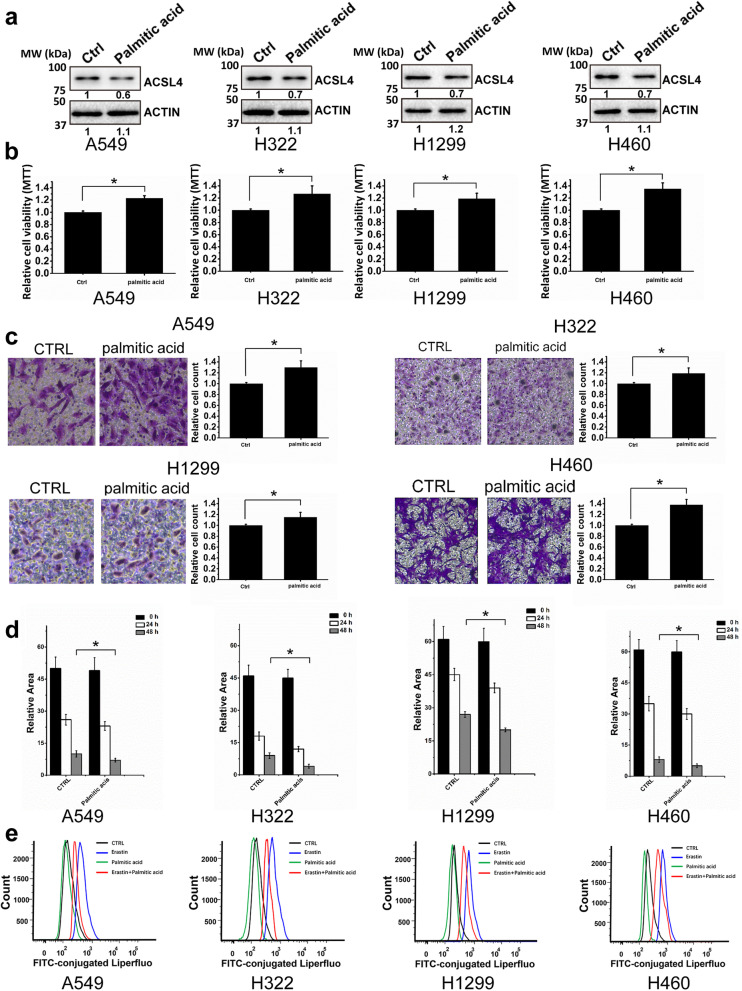
High**-**fat treatment promoted cell survival, invasion, and migration and inhibited ferroptosis. **a** The effect of palmitic acid treatment on ACSL4 expression in 4 different lung adenocarcinoma cell lines. Representative Western blot results were shown and ACTIN was used as the loading control. The relative blot intensity was listed below each band. **b** Cell viability was analyzed by MTT in 4 different lung adenocarcinoma cell lines treated with or without palmitic acid. **c** Cell migration and invasion were analyzed by the transwell assays in lung adenocarcinoma cells treated with or without palmitic acid. **d** Cell migration was analyzed by wound healing assay in lung adenocarcinoma cells treated with or without palmitic acid. **e** Liperfluo-based lipid peroxidation assay was used to monitor the ferroptosis levels in lung adenocarcinoma cells treated with erastin and/or palmitic acid

Previous studies suggest that lipid metabolism could affect ACSLs expression and cell sensitivity to ferroptosis [26, 27]. We thereby investigated the effects of high-fat treatment on lung adenocarcinoma. After culturing cells in high palmitic acid-containing media for 48 h, ACSL4 expression was decreased in all 4 lung adenocarcinoma cell lines (Fig. 5a). Consistent with the results in ACSL4 knockdown cells, palmitic acid-treated cells had an increased number of viable cells (Fig. 5b). Also, palmitic acid treatment promoted cell migration and invasiveness as measured by the transwell and wound healing assays (Fig. 5c,d, and Fig. S5). We then used erastin to induce ferroptosis in these cells. Strikingly, palmitic acid treatment could partially reverse the erastin-induced elevation of lipid peroxide, thus impairing the ferroptosis in lung adenocarcinoma cells (Fig. 5e).

**Fig. 6:**
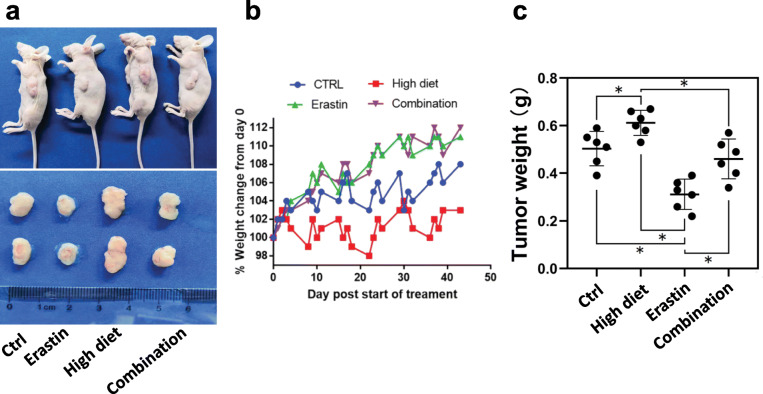
High diet reversed cell death caused by erastin in mice. **a** Representative images of mouse xenografts injected with A549 cells into the abdominal region. **b** The change in mouse body weight over time in the four mouse xenograft groups, including ordinary diet + PBS group (control group; n = 3), ordinary diet + erastin group (erastin group; n = 3), high-fat diet + PBS group (high-fat diet group; n = 3), and high-fat diet + erastin group (combination group; n = 3). **c** The final tumor weights were compared among four xenograft groups

Lastly, we studied the effect of the high-fat diet on ferroptosis in lung adenocarcinoma using the *in vivo* model. We injected A549 cells into the abdominal region of nude mice to generate lung adenocarcinoma mouse xenografts (Fig. 6a), and we then divided the 12 xenografts into 4 treatment groups, including ordinary diet + PBS group (control group; n = 3), ordinary diet + erastin group (erastin group; n = 3), high-fat diet + PBS group (high-fat diet group; n = 3), and high-fat diet + erastin group (combination group; n = 3). The high-fat diet group had the lowest body weight compared with other groups, implying that the high-fat diet was likely to worsen the disease progression of lung adenocarcinoma in mice (Fig. 6b). Similarly, the high-fat diet group had the highest tumor weight (Fig. 6c). Erastin treatment could significantly reduce the mouse tumor load, which was partially reversed by feeding mice with the high-fat diet (Fig. 6c). Therefore, both *in vitro* and *in vivo* results indicate that ferroptosis-inducing agents could inhibit the progression of lung adenocarcinoma and the high-fat diet could partially attenuate this anti-tumor effect by downregulating the tumor suppressor gene ACSL4.

## Discussion

Previous studies showed ACSL4 displayed both tumor-promoting and tumor-suppressive functions in different tumor types, and the role of ACSL4 in lung adenocarcinoma was still elusive. In the current study, we performed a serial of experiments to assess the function of ACSL4 in lung adenocarcinoma cells. Interestingly, our results were similar to the studies of ACSL4 in gastric cancer tissues. ACSL4 was also a promising prognostic factor in lung adenocarcinoma. For patients with low ACSL4 expression, more active adjuvant chemotherapy, radiotherapy, targeted therapy, or even immunotherapy may be given. Moreover, considering the anti-tumor effect of ACSL4, it may be a new therapeutic target for the targeted therapy of lung adenocarcinoma.

ACSL4 expression level has been studied in most cancer types, and ACSL4 becomes a promising drug target for certain cancers [28–30]. Chromophobe renal cell carcinoma, renal cell carcinoma, tubular cell carcinoma, lung adenocarcinoma, and lung squamous carcinoma showed lower ACSL4 expression, while cholangiocarcinoma, colon cancer, and hepatic cell carcinoma showed higher expression level. ACSL4 upregulation in breast cancer, liver cancer, and colorectal cancer was reported to be related to recurrence and metastasis, and inhibiting ACSL4 in liver cancer cells attenuated cell growth [31]. In contrast, ACSL4 plays a tumor-suppressive role in gastric cancer and was thus frequently downregulated [28], and forced ACSL4 overexpression in gastric cancer cells impaired cell growth and migration [28], which is similar to what we have observed in lung adenocarcinoma cells. The mechanism underlying this context-dependent role of ACSL4 in different cancer types is still elusive. It is known that ACSL4 catalyzes a broad range of fatty acid substrates, and its function in fatty acid metabolism is also cell type-dependent [32]. Given that fatty acid metabolism is widely involved in tumorigenesis and cancer progression [33], ACSL4 might catalyze different kinds of fatty acid substrates in different cancer types, thereby re-programming the cell metabolomics to be either tumor-promoting or tumor-inhibiting. Nevertheless, the precise role of ACSL4 in cancer still needs to be carefully investigated in future studies.

Ferroptosis is a programmed cell death process characterized by the accumulation of iron-dependent lipid peroxides. Erastin-induced ferroptosis through GPX4 inactivation played an important role in inhibiting tumor growth and killing tumor cells. Related therapies have been reported in hepatocellular carcinoma, renal cell carcinoma, non-small cell lung cancer, ovarian cancer, pancreatic cancer, and diffuse large B-cell lymphoma [5–7, 34–37]. Ferroptosis was also associated with sensitivity to chemotherapy drugs and immunotherapy [22, 38–41]. A high level of lipid peroxides in lung cancer tissues suggested the possibility of ferroptosis in lung cancer. In order to avoid cell death, lung cancer cells use several ways to increase the induction threshold of ferroptosis, such as upregulating system xc-, increasing the antioxidant capacity, maintaining GPX4 activity, and inducing lymphoid-specific helicase to regulate lipid metabolism [42–44]. Our data confirmed that ACSL4 expression positively regulated response to oxidative stress pathway in lung adenocarcinoma and negatively correlated with GPX4 in ferroptotic cell death signaling pathways. This may hint that small molecules such as erastin and RSL3 had the potential efficacy to kill tumor cells. At present, the markers of ferroptosis include iron level, lipid peroxidase (LPO) level, GPX4 activity, GSH content, ACSL4 activity, SLC7A11 expression, NOX1 expression, and cell morphology using electron microscopy. In our study, we mainly used liperfluo to monitor ferroptosis and detect lipid hydroperoxides in living cells. Among fluorescent probes that detect reactive oxygen species (ROS), liperfluo is the only compound that can specifically detect lipid peroxides by flow cytometric analysis, and liperfluo showed high sensitivity and specificity for monitoring ferroptosis [45]. Furthermore, using both the cell line model and mouse xenograft model, we found that high-fat treatment could downregulate ACSL4 expression in lung adenocarcinoma cells, thus enhancing cell survival and migration and reducing ferroptotic cell death. High-fat treatment reduced the effect of ferroptotic cell death induced by erastin. Agents to induce ferroptosis may be an effective strategy in the treatment of lung adenocarcinoma without the high-fat diet. Our study provided new theoretical evidence for the application of ferroptotic inducers in the treatment of lung adenocarcinoma and dietary guidance for the patients.

Our study also had several limitations, including limited clinical samples and a lack of ferroptotic markers. Future studies are needed to completely understand the functional difference of ACSL4 between lung adenocarcinoma and other cancer types. Also, the effects of silencing or overexpression of ACSL4 on cell viability were relatively mild in some of the lung adenocarcinoma cell lines, suggesting that the expression level of ACSL4 may be only one of the factors that affect the tumor viability in lung adenocarcinoma and some other oncogenic pathways (e.g., EGFR pathway) may also play an important roles in tumor maintenance and progression.

## Conclusions

In summary, this is the first study demonstrating ACSL4 acts as a tumor suppressor in lung adenocarcinoma. Functional assays indicated that high**-**fat treatment promoted cell survival, invasion, and migration. Our study provided new evidence that the high-fat diet suppresses ferroptosis via down-regulating ACSL4 in lung adenocarcinoma.

### Supplementary Information

**Additional file 1: Fig. S1.** Aberrant ACSL4 expression in different types of cancers. The ACSL4 expression data in the tumors (shown in red) and the corresponding normal tissues (shown in grey) were obtained from the TCGA database, and the number of tumor and normal samples (num (T) and num (N), respectively) was shown below each boxplot. The red asterisk represents p value<0.05. BRCA, breast invasive carcinoma; CHOL, cholangial carcinoma; COAD, colon adenocarcinoma; KICH, Kidney Chromophobe; KIRC, kidney renal clear cell carcinoma; KIRP, kidney renal papillary cell carcinoma; LIHC, liver hepatocellular carcinoma; PRAD, prostate adenocarcinoma.

**Additional file 2: Fig. S2.** Wound healing assay of lung adenocarcinoma cell lines in response to ACSL4 knockdown or overexpression.

**Additional file 3: Fig. S3. **Ferroptosis has an impact on cell survival, invasion, and migration in adenocarcinoma cell lines.aWound healing assay of lung adenocarcinoma cell lines treated with PBS control (Ctrl), erastin, or Ferrostatin-1. The multiple comparisons of student’s t-test (two-tailed) were corrected using the Bonferroni method (n=3; *p<0.05, **p<0.01, ***p<0.001). b Cell migration and invasion were analyzed by the transwell assays in lung adenocarcinoma cells treated with either erastin or Ferrostatin-1 (n=3; *p<0.05, **p<0.01, ***p<0.001). c Cell viability was analyzed by MTT in 4 different lung adenocarcinoma cell lines treated with either erastin or Ferrostatin-1 (n=3; *p<0.05, **p<0.01, ***p<0.001).

**Additional file 4: Fig. S4. **Representative FACS results depicting apoptosis of human A549 cells (control, shACSL4, or oeACSL4) treated with or without erastin (2 μM) for the indicated times. H_2_O_2_ (10mM) treatment in A549 cells was used as the positive control for apoptosis.

**Additional file 5: Fig. S5.** Wound healing assay of lung adenocarcinoma cell lines treated with or without palmitic acid.

**Additional file 6.**

## Data Availability

The data that support the findings of this study are available on request from the corresponding author.
