# Correction: High-fat diet impairs ferroptosis and promotes cancer invasiveness via downregulating tumor suppressor ACSL4 in lung adenocarcinoma

**DOI:** 10.1186/s13062-023-00419-0

**Published:** 2023-10-26

**Authors:** Yixiang Zhang, Songyu Li, Fengzhou Li, Changsheng Lv, Qing-kai Yang

**Affiliations:** 1 Department of Thoracic Surgery, The First Affiliated Hospital of Dalian Medicine University, No. 222 Zhongshan Road, Dalian, Liaoning 116000 China; 2 Department of Oncology, Institute of Cancer Stem Cell, Dalian Medical University, 9 Western Lvshun South Road, Dalian, Liaoning 116044 China

After publication of this article [1], it was brought to our attention that Fig. 5 need to be corrected, the correct figure is provided below.


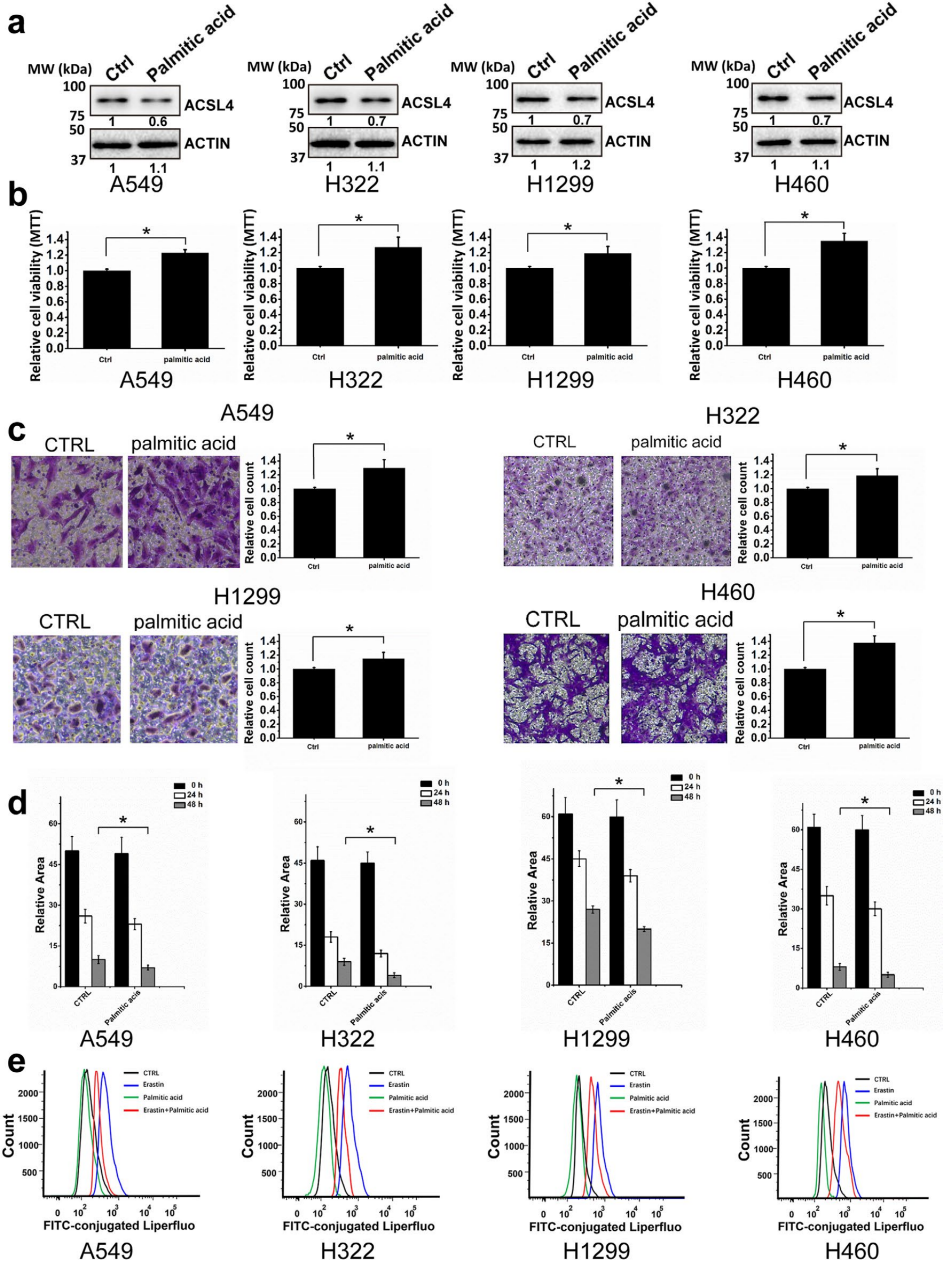


The original publication has been corrected.
