# Supplementary figures and images for: High-fat diet impairs ferroptosis and promotes cancer invasiveness via downregulating tumor suppressor ACSL4 in lung adenocarcinoma
Source: Biol Direct. 2021 May 31;16:10. doi: 10.1186/s13062-021-00294-7 (PMC8166005; doi:10.1186/s13062-021-00294-7)

Figure S1

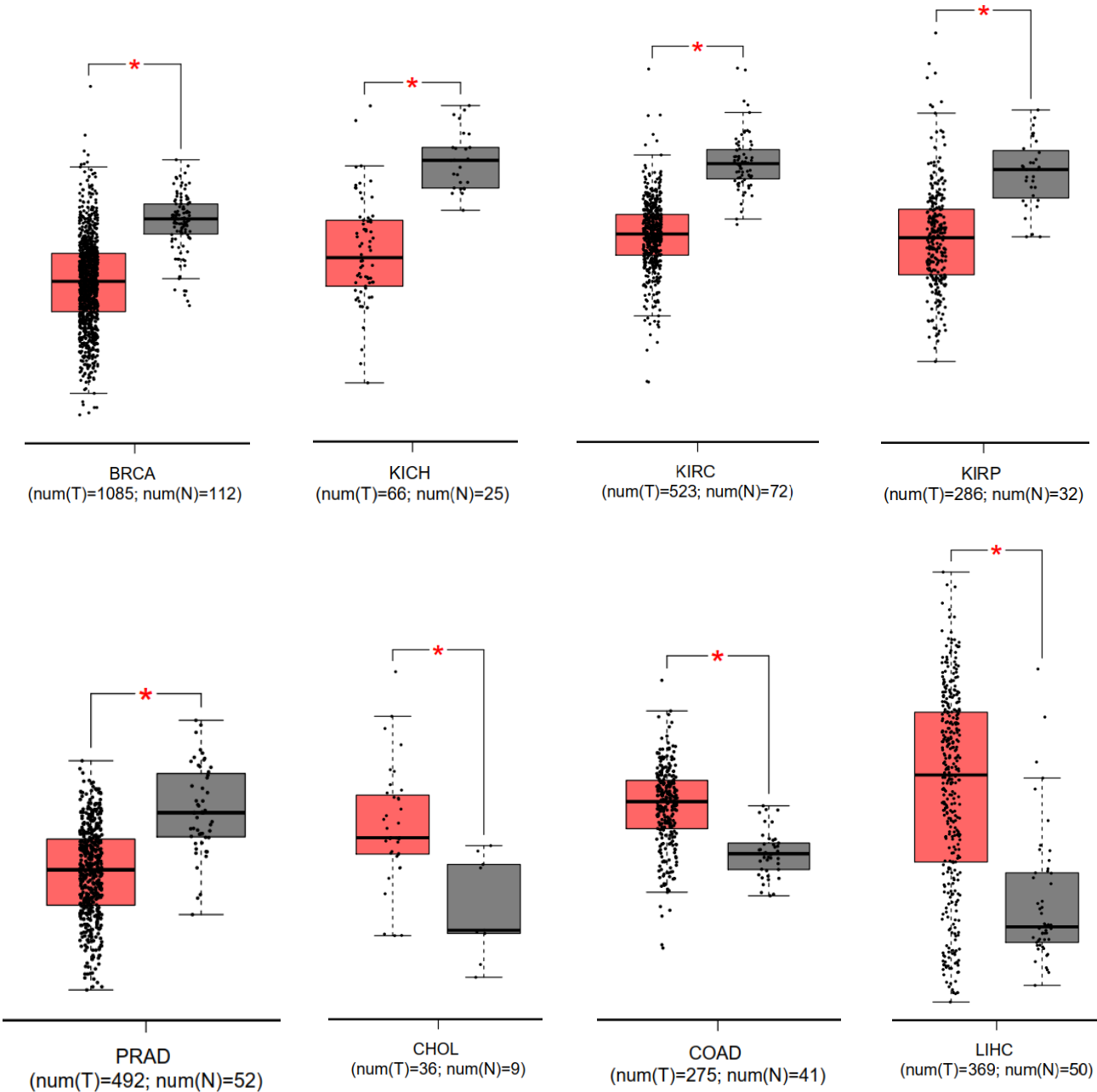

Supplement: Supplementary file 1 — Additional file 1: Fig. S1. Aberrant ACSL4 expression in different types of cancers. The ACSL4 expression data in the tumors (shown in red) and the corresponding normal tissues (shown in grey) were obtained from the TCGA database, and the number of tumor and normal samples (num (T) and num (N), respectively) was shown below each boxplot. The red asterisk represents p value<0.05. BRCA, breast invasive carcinoma; CHOL, cholangial carcinoma; COAD, colon adenocarcinoma; KICH, Kidney Chromophobe; KIRC, kidney renal clear cell carcinoma; KIRP, kidney renal papillary cell carcinoma; LIHC, liver hepatocellular carcinoma; PRAD, prostate adenocarcinoma. [file 13062_2021_294_MOESM1_ESM.pdf]

Figure S2

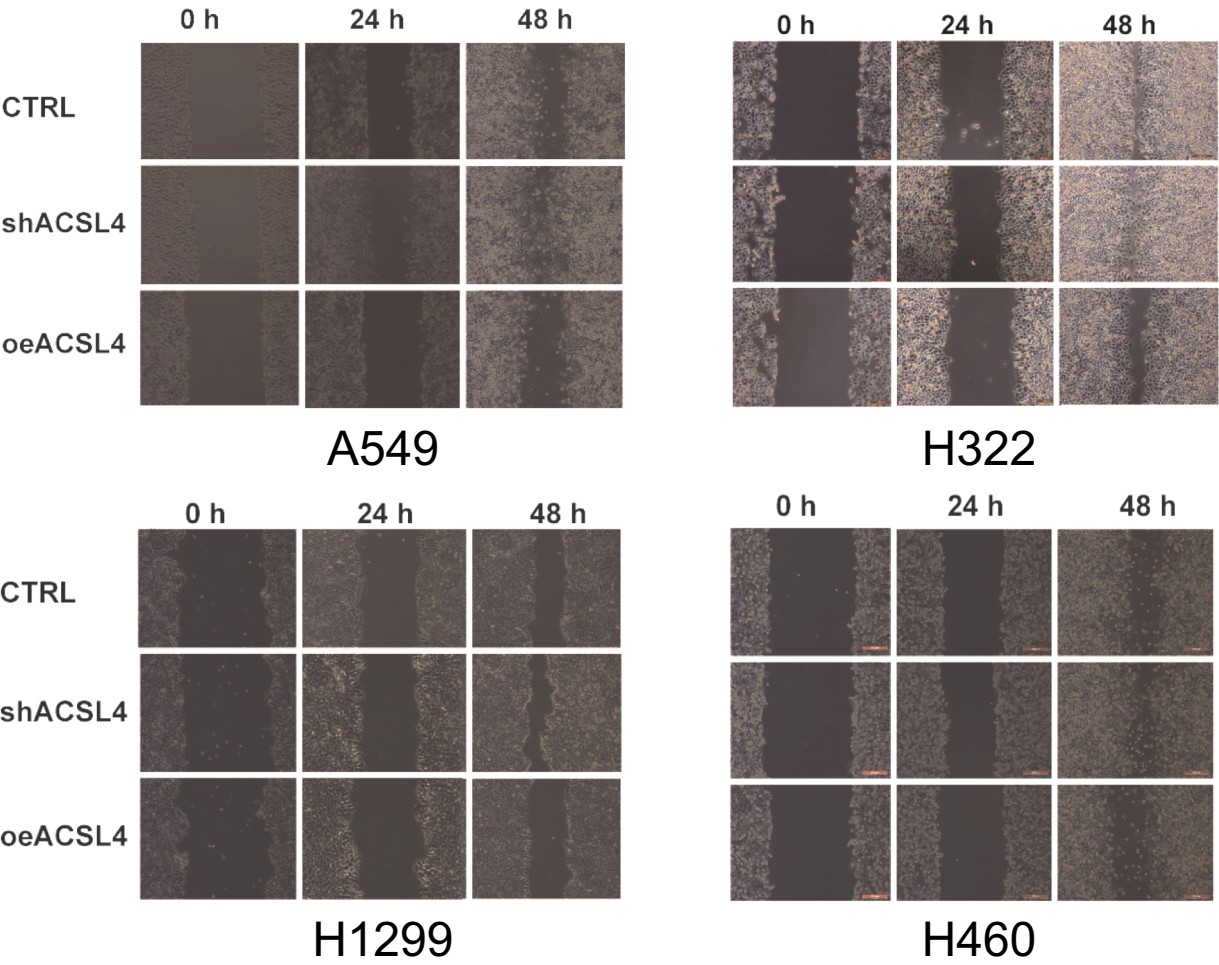

Supplement: Supplementary file 2 — Additional file 2: Fig. S2. Wound healing assay of lung adenocarcinoma cell lines in response to ACSL4 knockdown or overexpression. [file 13062_2021_294_MOESM2_ESM.pdf]

Figure S3

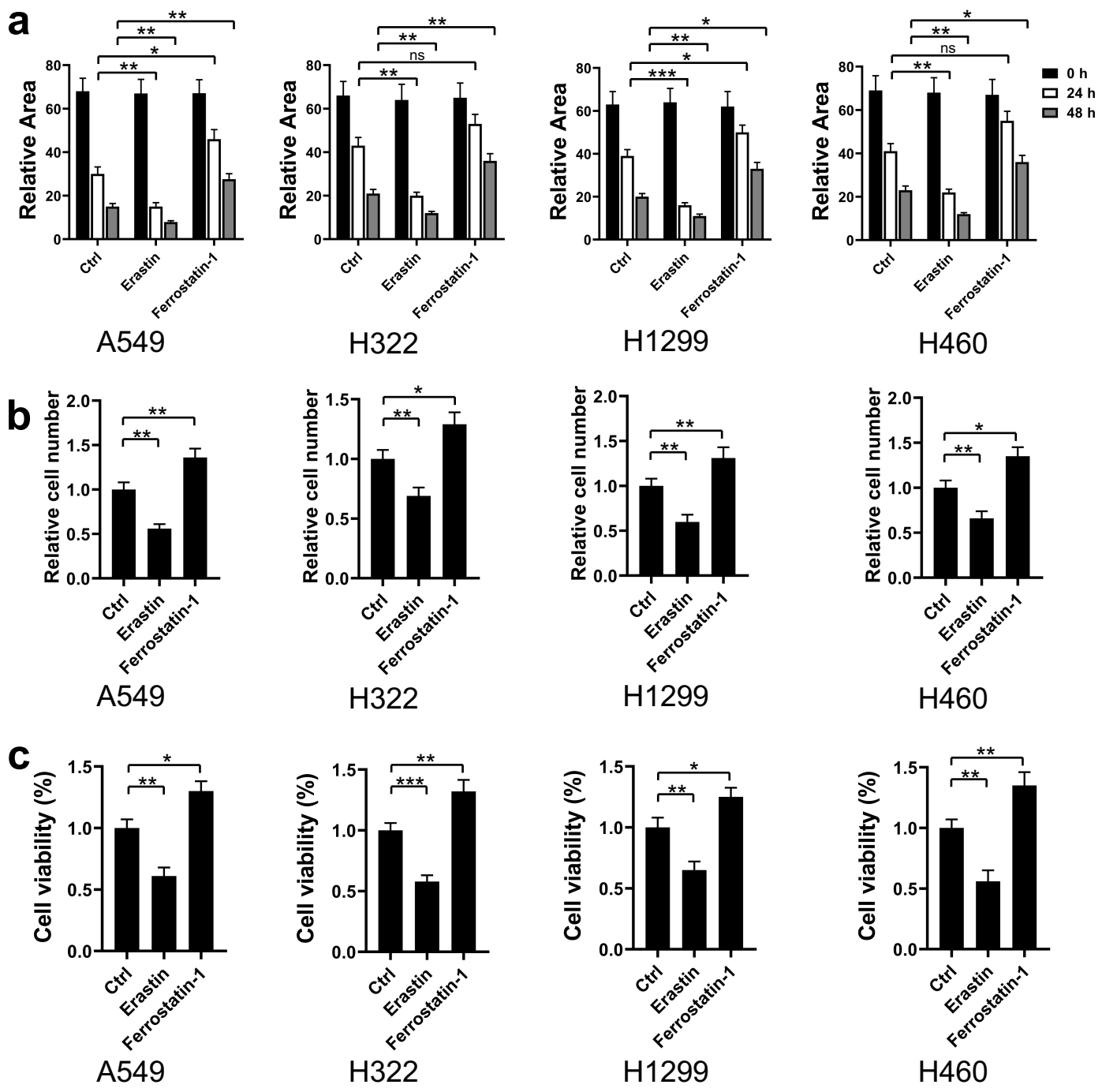

Supplement: Supplementary file 3 — Additional file 3: Fig. S3. Ferroptosis has an impact on cell survival, invasion, and migration in adenocarcinoma cell lines.aWound healing assay of lung adenocarcinoma cell lines treated with PBS control (Ctrl), erastin, or Ferrostatin-1. The multiple comparisons of student’s t-test (two-tailed) were corrected using the Bonferroni method (n=3; *p<0.05, **p<0.01, ***p<0.001). b Cell migration and invasion were analyzed by the transwell assays in lung adenocarcinoma cells treated with either erastin or Ferrostatin-1 (n=3; *p<0.05, **p<0.01, ***p<0.001). c Cell viability was analyzed by MTT in 4 different lung adenocarcinoma cell lines treated with either erastin or Ferrostatin-1 (n=3; *p<0.05, **p<0.01, ***p<0.001). [file 13062_2021_294_MOESM3_ESM.pdf]

Figure S4

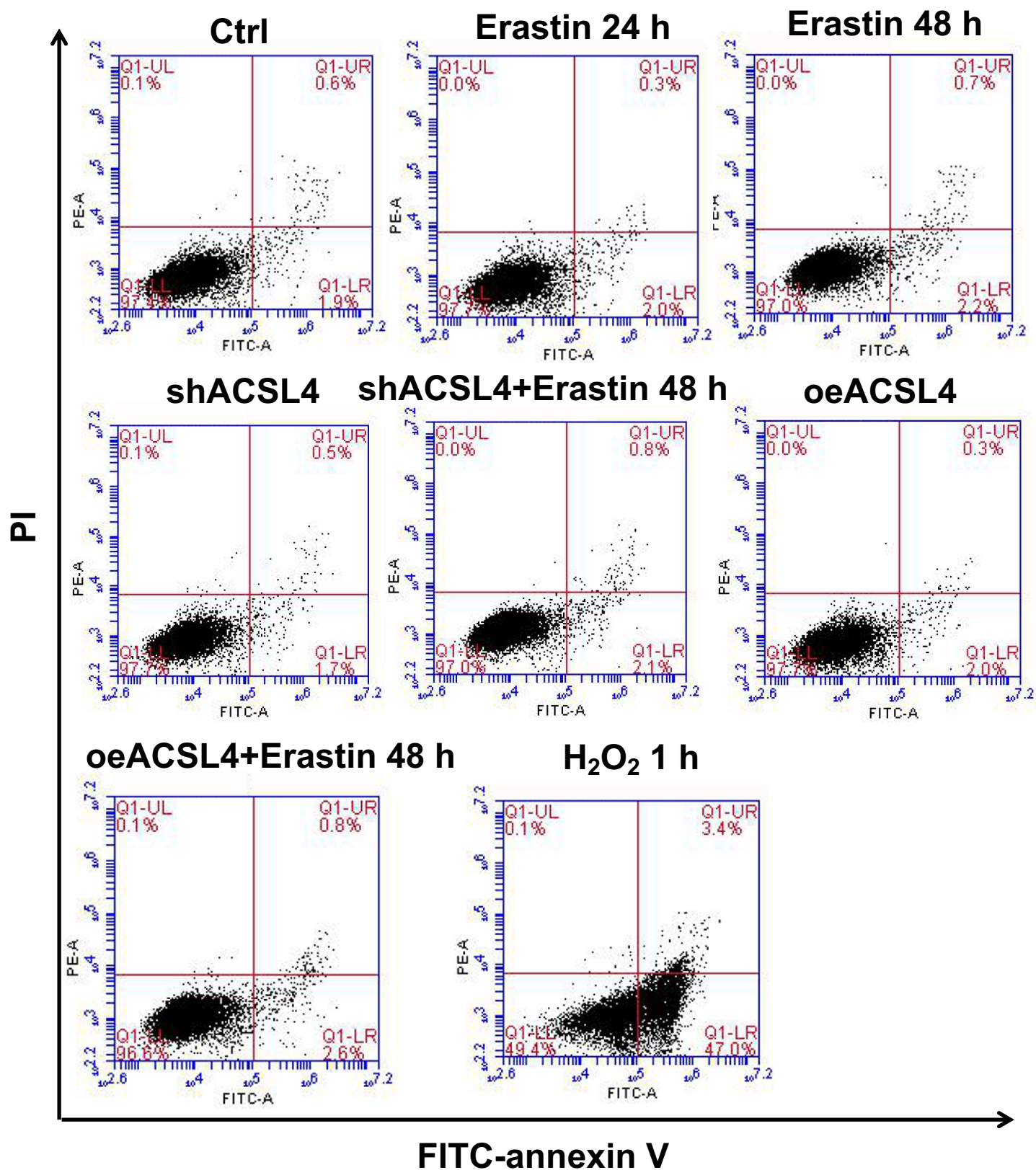

Supplement: Supplementary file 4 — Additional file 4: Fig. S4. Representative FACS results depicting apoptosis of human A549 cells (control, shACSL4, or oeACSL4) treated with or without erastin (2 μM) for the indicated times. H2O2 (10mM) treatment in A549 cells was used as the positive control for apoptosis. [file 13062_2021_294_MOESM4_ESM.pdf]

Figure S5

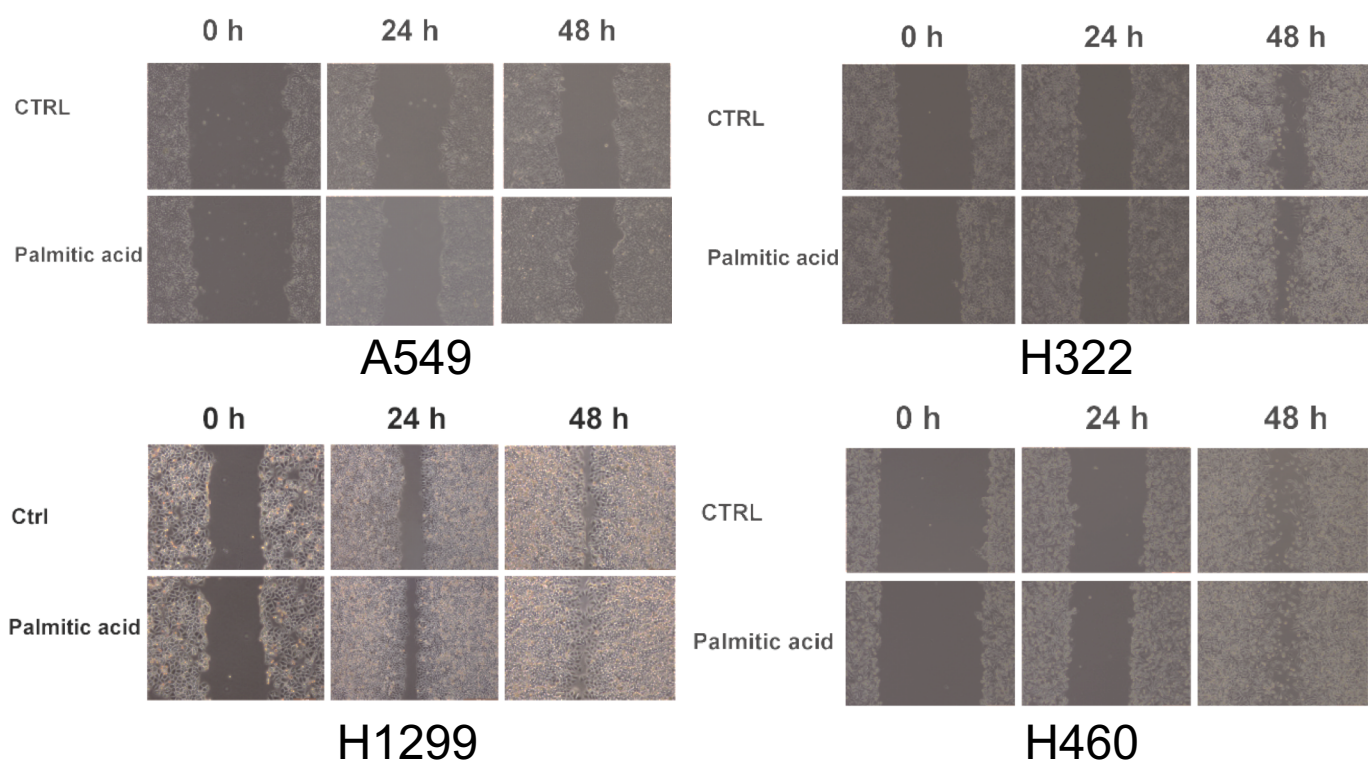

Supplement: Supplementary file 5 — Additional file 5: Fig. S5. Wound healing assay of lung adenocarcinoma cell lines treated with or without palmitic acid. [file 13062_2021_294_MOESM5_ESM.pdf]
